# P-404. Overnight Meropenem Approval at Stand-Alone Children's Hospital

**DOI:** 10.1093/ofid/ofaf695.621

**Published:** 2026-01-11

**Authors:** Victoria A Hellner, Grant Stimes, Debra Palazzi

**Affiliations:** Texas Children's Hospital, Houston, Texas; Texas Children's Hospital, Houston, Texas; Baylor College of Medicine, Houston, TX

## Abstract

**Background:**

Meropenem (MEM) is used in pediatric patients for the treatment of serious infections due to multi drug-resistant organisms (MDRO). Prior-authorization is often required by antimicrobial stewardship programs for MEM use. At TCH, overnight infectious diseases (ID) approvals for MEM increased significantly from 2020-2023 raising concerns about increasing approving-physician burden. Concern for physician wellness and duty hour implications of frequent overnight calls let to a system-wide policy change beginning in 11/2023. This change allowed for a one-time MEM dose between 22:00 and 06:00 without ID approval. After 06:00, the primary team consulted ID to continue therapy. The objective of this study was to evaluate policy adherence.

**Methods:**

This retrospective chart review was conducted at Texas Children’s Health System containing 1 large quaternary pediatric hospital and 3 community hospitals. Patients were eligible for inclusion if they received a dose of MEM between 22:00 and 06:00 from 11/16/2023-9/30/2024.

The primary outcome was number of orders correctly following the policy. Secondary outcomes included MEM continued after the once dose and time to next dose of effective antibiotic. Data collected included demographics, time of MEM dose, date and time of next antibiotic dose, renal function, history of MDRO in the prior 180 days, comorbidities, and resolution/recurrence of infection.

**Results:**

56 courses of MEM were ordered overnight; 26 (46%) were for patients with a history of MDRO requiring MEM. Four (7.1%) courses were for definitive therapy rather than empiric use. The median time to next dose of antibiotic after MEM was 8.68 hours (within 1 dosing interval). Six courses did not follow policy by ordering more than 1 dose of MEM during the 22:00-06:00 timeframe.

**Conclusion:**

Overnight approval of once doses of MEM largely followed policy. Overnight approvals of antimicrobials outside of normal antibiotic stewardship pre-authorizations may address physician wellness and duty hour concerns without significantly promoting overuse of restricted agents.

**Disclosures:**

Debra Palazzi, MD, MEd, AHRQ: Grant/Research Support|AMA: Board Member|AMA: JAMA Pediatrics Associate Editor|Elsevier: UpToDate|PEW Charitable Trust: Grant/Research Support|University of Chicago/Lurie Children's: Honoraria

